# Serotonin Transporter Gene Polymorphism Modulates Activity and Connectivity within an Emotional Arousal Network of Healthy Men during an Aversive Visceral Stimulus

**DOI:** 10.1371/journal.pone.0123183

**Published:** 2015-04-20

**Authors:** Lisa A. Kilpatrick, Emeran A. Mayer, Jennifer S. Labus, Arpana Gupta, Toyohiro Hamaguchi, Tomoko Mizuno, Hazuki Komuro, Michiko Kano, Motoyori Kanazawa, Masashi Aoki, Shin Fukudo

**Affiliations:** 1 Oppenheimer Family Center for Neurobiology of Stress, Division of Digestive Diseases, David Geffen School of Medicine at University of California at Los Angeles (UCLA), Los Angeles, California, United States of America; 2 Department of Behavioral Medicine, Tohoku University Graduate School of Medicine, Sendai, Japan; 3 Department of Psychosomatic Medicine, Tohoku University Graduate School of Medicine, Sendai, Japan; 4 Department of Neurology, Tohoku University Graduate School of Medicine, Sendai, Japan; University of L'Aquila, ITALY

## Abstract

**Background and Aims:**

The 5-hydroxytryptamine transporter gene-linked polymorphic region (5-HTTLPR) has been linked to increased stress responsiveness and negative emotional states. During fearful face recognition individuals with the *s* allele of 5-HTTLPR show greater amygdala activation. We aimed to test the hypothesis that the 5-HTTLPR polymorphism differentially affects connectivity within brain networks during an aversive visceral stimulus.

**Methods:**

Twenty-three healthy male subjects were enrolled. DNA was extracted from the peripheral blood. The genotype of 5-HTTLPR was determined using polymerase chain reaction. Subjects with the *s/s* genotype (n = 13) were compared to those with the *l* allele (genotypes *l/s*, *l/l*, n = 10). Controlled rectal distension from 0 to 40 mmHg was delivered in random order using a barostat. Radioactive H2[^15-^O] saline was injected at time of distension followed by positron emission tomography (PET). Changes in regional cerebral blood flow (rCBF) were analyzed using partial least squares (PLS) and structural equation modeling (SEM).

**Results:**

During baseline, subjects with *s/s* genotype demonstrated a significantly increased negative influence of pregenual ACC (pACC) on amygdala activity compared to *l*-carriers. During inflation, subjects with *s/s* genotype demonstrated a significantly greater positive influence of hippocampus on amygdala activity compared to *l*-carriers.

**Conclusion:**

In male Japanese subjects, individuals with *s/s* genotype show alterations in the connectivity of brain regions involved in stress responsiveness and emotion regulation during aversive visceral stimuli compared to those with *l* carriers.

## Introduction

The serotonin (5-hydroxytryptamine; 5-HT) signaling system plays an important role in modulating a wide range of brain functions [1], including brain networks involved in emotion regulation, stress response and processing of interoceptive signals [2]. In the human brain, 5-HT is produced in the 5-HT neurons which originate from the raphe nuclei in the brain stem, and released 5-HT has pre- and post-synaptic effects via a wide range of 5-HT receptors [1]. Once released, 5-HT is taken up by neurons and platelets via the 5-HT transporter (5-HTT) and other transporters [3] and is metabolized by monoamine oxydase A (MAOA) in the mitochondria [1]. The concentration of 5-HT in the synaptic cleft is largely determined by the 5-HTT. 5-HTT has been implicated as neuromarker related to memory, amnesia, and drug effects [4].

5-HTT is encoded by the human 5-HTT gene (SLC6A4), which is located on chromosome 17q12 [5]. A variant in the upstream promoter region of the 5-HTT gene has been identified [5]. This 5-HTT linked polymorphic region (5-HTTLPR) with long (*l*, 528 bp) and short (*s*, 484 bp) forms affects the expression and function of 5-HTT, the *s* allele being associated with lower transcriptional efficiency of the promoter than the *l* allele [5]. Reduced expression of 5-HTT results in a higher synaptic 5-HT concentration and down regulation of pre- and post-synaptic 5-HT receptors [6]. Such alterations in the serotonergic synapse may acutely alter the response of the brain to certain stimuli, or it may affect the role of 5-HT during brain development [7] or both. Such changes in 5-HT signaling have been implicated in the increased susceptibility to negative mood states, and increased stress responsiveness in individuals with the *s* allele. Consistent with this model, epidemiological evidence has shown that individuals with the *s* allele are at significantly greater risk for major depressive disorder following repeated stress in adult or trauma in childhood [8] and these findings were strongly supported by a recent meta-analysis with a larger sample [9]. Individuals with the *s* allele also have been found to show increased fear and anxiety-related behaviors and greater amygdala neuronal activity as assessed by blood oxygen level-dependent functional magnetic resonance imaging (fMRI) when viewing fearful faces compared to individuals homozygous for the *l* allele [10]. Analysis of functional connectivity of the anterior cingulate cortex (ACC) and amygdala during perceptual processing of fearful stimuli demonstrated tight coupling as a feedback circuit implicated in the extinction of negative affect and *s* allele carriers showed relative uncoupling of this circuit [11].

The relationship between 5-HTTLPR and emotional regulatory circuitry in the brain may depend on sex and/or ethnicity. For example, the 5-HTTLPR genotypes in Japanese were previously reported to be 3% *l/l*, 28% *l/s*, and 68% *s/s* [12]. By contrast, those in multiethnic samples in the US were 32% *l/l*, 49% *l/s*, and 19% *s/s*. Because the prevalence of diagnosed anxiety disorder (18.1%) and mood disorder (9.5%) in the western countries [13] is greater than that of corresponding prevalence numbers in Japan (anxiety disorder, 1.8%; mood disorder, 4.1%) [14], the traditional concept of a simple relationship between 5-HTTLPR genotype and risk for disorders of negative emotion is questionable. Mizuno et al. [15] demonstrated that among Japanese individuals, males with *s/s* genotype showed higher anxiety than those with *l/s* genotype while females displayed the opposite pattern with higher anxiety in *l/s* compared to *s/s* females, suggesting that Japanese women may not have the same relation between 5-HTTLPR and negative emotion as reported in Western studies. Probably it is due to gene-sex-environment interaction [15].

In our previous study with a mixed sample of predominantly male Japanese healthy control subjects (HCs) and patients with irritable bowel syndrome (IBS), all individuals with *s/s* genotype, showed a larger increase in regional cerebral blood flow (rCBF) of the ACC, parahippocampal gyrus, and orbitofrontal cortex (OFC) in the context of aversive colorectal distention compared to those with the *l* allele [16]. In the current study, we aimed to test the hypothesis that healthy male Japanese individuals with *s/s* genotype differ from *l* carries in the connectivity of brain networks engaged by aversive visceral stimulation.

## Subjects and Methods

### Subjects

Twenty-three healthy adult male Japanese subjects without organic diseases or psychiatric disorders participated in the study. Age of the subject ranged from 20 yo to 26 yo. Psychiatric disease was excluded through an unstructured clinical interview conducted by a board-certified specialist of the Japanese Society of Psychosomatic Medicine and via a structured interview using the Structured Clinical Interview [17] for Diagnostic and Statistical Manual of Mental Disorders, 4th Edition, Text Revision [18]. All subjects were right-handed. This study was performed in accordance with the ethical standards laid down in the 1964 Declaration of Helsinki and its later amendments and approved by the Ethics Committee of Tohoku University Graduate School of Medicine (#2012-1-379). All subjects provided written informed consent. The subjects were independent from those in our previously published study [16].

### Genotyping

On the day of experiment, a plastic catheter was inserted into the left forearm vein of each subject and saline was infused at a speed of 1.6 ml/min. Peripheral blood was sampled with a heparinized syringe. Genotyping was performed using the same methods as in our previous reports [15, 16]. In brief, DNA was extracted from lymphocytes. The polymorphism in the regulatory region of the 5-HTT gene was genotyped by polymerase chain reaction (PCR). PCR-amplification was carried out using primer pairs reported by Lesch et al. [5] (5’-GGC GTT GCC GCT CTG AAT GC-3’ and 5’-GAG GGA CTG AGC TGG ACA ACC AC-3’). A 25 microl PCR reaction consisted of a 0.2 microM concentration of each primer, 1.5 mM MgSO_4_, 0.2 mM each of deoxynucleotide triphosphate, 1X PCR_X_ Amplification Buffer, 2.5 U of PLATINUM Taq DNA Polymerase, and 1X PCR_x_ Enhancer Solution (GIBCO BRL, Life Technologies Inc., Rockville, MD, USA). After initial denaturation at 95°C for 2 minutes, amplification was performed using 35 cycles at 95°C for 30 s, 60°C for 30 s (annealing), and 68°C for 1 minute, followed by a final elongation at 68°C for 3 minutes. The amplification products were separated on 2% agarose gel by electrophoresis and classified as long and short alleles.

To ensure genotype accuracy, sequence analysis of 5-HTTLPR genes was performed on PCR fragments, which were amplified according to the previously described protocol. PCR products were purified from agarose gel using a QIAquick Gel Extraction kit (QIAGEN, Hilden, Germany). Amplimers were sequenced directly using the ABI PRISM dRodamine Terminator Cycle Sequencing Ready Reaction kit (PE Applied Biosystems, Foster City, CA, USA), and excess dye terminators were removed using CENTRI-SEP Columns (PRINCETON SEPARATIONS, Adelphia, NJ, USA). Automated sequencing was performed on an ABI 310 Genetic Analyzer (PE Applied Biosystems). All procedures were performed according to manufacturer’s instructions. Forward and reverse primers were used to sequence the PCR products.

### Visceral Stimulation

Colorectal stimulation was performed using the same methods as previously described [16, 19]. On the day before the experiment, subjects were given low-residue meals and their colorectum was cleansed. On the experimental day, a catheter with a barostat bag (700 ml in volume) was inserted into the rectum. Colorectal distention stimuli were provided with a computerized barostat equipment (Medtronics Synectics, Shoreview, MN, USA), which inflated the bag at a rate of 38 ml/s. The colorectum was stimulated with bag pressures of 0 mmHg (non-inflation: non-INF) or 40 mmHg (inflation: INF) for 80 s. The intensity of each stimulus was randomly chosen to avoid stimulation order effects, and the time interval between two stimuli was 15 min.

### Brain imaging

Scans of the distribution of H_2_^15^O were obtained using a SET-2400W PET scanner (Shimadzu, Japan) operated on a high sensitivity three-dimensional mode with an average axial resolution of 4.5 mm at maximum strength and sensitivity for a 20-cm cylindrical phantom of 48.6k.c.p.s.kBq-1ml-1 [20, 21]. For each scan, a subject received approximately 5 mCi (185 MBq) of H_2_^15^O intravenously through the forearm vein and underwent colorectal distention during rCBF measurement. The radioactivity peak to the scan onset was about 10 s after the start of colorectal distention at which both the radioactivity peak and peak pressure of the bag simultaneously reached a plateau. The PET scanning room was darkened and the subjects, while awake, were instructed to keep their eyes closed for the whole period of scanning (70 s).

### Image Preprocessing

Statistical parametric mapping software (SPM2, Wellcome Department of Cognitive Neurology, London, UK) was used for PET image realignment, normalization, smoothing, and to create statistical maps of significant rCBF changes [22, 23]. All rCBF images were stereotaxically normalized into the standard space defined by Talairach and Tournoux [24] using an rCBF template image supplied within SPM2. The normalized images were smoothed using a 12×12×12-mm Gaussian filter, and the rCBF values were expressed in ml dl−1 min−1, adjusted for individual global CBF values using ANCOVA, and scaled to a mean of 50.

### Statistical Analyses

Partial least squares (PLS) is a multivariate statistical technique considered to be more sensitive than standard univariate analyses of neuroimaging data such as SPM [25, 26]. PLS is analogous to principal components analysis (PCA), but the solutions can be restricted to the part of the covariance structure that is attributable to groups in an experimental design. A task PLS analysis was employed to identify distributed patterns of regions associated with rectal balloon inflation in healthy men with *s/s* genotype and *l*-carriers (*l/s* and *l/l* genotypes). PLS was implemented with freely available code (http://www.rotman-baycrest.on.ca). Voxel reliability was determined using bootstrap estimation (500 samples). The ratio of the observed weight to the bootstrap standard error was calculated and voxels were considered reliable if the absolute value of the bootstrap ratio (BSR) exceeded 3.23 (p < 0.001). Clusters greater than 25 voxels are reported. All functional data were overlaid on the MNI template available in MRIcron (http://www.cabiatl.com/mricro/mricron/index.html) for presentation purposes.

Structural equation modeling (SEM) was applied to simultaneously quantify the interactions among brain regions and to test for genotype differences in the effective connectivity of a hypothesized network [26]. SEM requires a priori specification of a structural (anatomical) model for testing. The hypothesized network was based on well-recognized brain networks of visceral perception from our laboratories [2, 16, 19–21, 27]. The nodes of the network were chosen from right hemisphere clusters with a |BSR| >1.96 (p < 0.05) in the task PLS. Normalized activity from each scan was extracted from the most reliable voxel of each region selected as a node of the network and entered into SEM. The connections among the brain regions comprising the network was supported by previous neuroanatomical studies [28]. Although connectivity between regions is often reciprocal, there are mathematical restrictions on the number of reciprocal pathways that can be specified for a given model using SEM [29]. Thus, we modeled most paths as unidirectional. SEM was performed using Amos 6.0. Residual variances representing external input into the system (e.g., unspecified regions, psychological characteristics) were fixed at 35% of the observed regional variances within group and condition [30]. SEM computes a path coefficient that represents the coupling between regions. Path coefficients reflect how a one unit change in one area influences activity in the region to which it projects, controlling for all other regions in the model.

Genotype differences in the effective connectivity of the network were tested using multi-group tests for invariance [31, 32]. Pair-wise comparisons were made between a completely unconstrained model and a partially constrained model in which a path of interest was restricted to be equal across genotype. Chi-square statistics for group differences and critical ratios for path coefficients were considered significant at p < 0.05.

## Results

### Genotyping

Thirteen subjects were homozygous for the *s* allele, 7 subjects carried the heterozygous *s/l* genotype, and 3 subjects were homozygous for the *l* allele. Subjects carrying an *l* allele were combined into a single group (“*l*-carriers”) for analyses (n = 10).

### Task PLS

Task PLS revealed a significant network of regions (p < 0.001, 66% cross-covariance matrix variance) differentiating INF from non-INF. Compared to *l*-carriers, men with *s/s* genotype showed greater engagement of this network, which included increased activation of regions of a sensory network (including bilateral thalamus, right anterior/mid insula, basal ganglia), increased activation of prefrontal and parietal cortical regions, and deactivation of regions of an affective network (amygdala, parahippocampal gyrus, hippocampus and subgenual ACC [sgACC] during INF (Table 1).

**Table 1:** Activations and deactivations within a network distinguishing inflation from non-Iinflation that is engaged to a greater extent in subjects with *s/s* genotype compared to *l*-carriers.

Region	Hemisphere	X	Y	Z	BS ratio	p	Cluster size
**ACTIVATIONS**							
Insula	right	42	6	2	4.61	<.001	197
	left	-32	-42	16	7.64	<.001	59
Thalamus	right	8	-28	14	8.09	<.001	626
	left	-14	-24	16	4.72	<.001	155
Putamen	left	30	-6	6	3.85	<.001	30
	right	-26	0	12	5.41	<.001	146
Caudate	right	18	6	20	5.39	<.001	132
Prefrontal cortex							
BA 11	right	26	52	-20	4.19	<.001	26
BA 46	right	48	46	18	4.42	<.001	135
BA 47	right	30	38	-4	5.71	<.001	192
BA 9	left	-50	18	30	4.94	<.001	56
BA 6	right	10	-22	50	4.73	<.001	75
Parietal cortex							
BA 40	left	-64	-54	30	5.21	<.001	55
	right	72	-48	24	5.30	<.001	71
BA 7	right	2	-84	42	5.85	<.001	55
Cerebellum							
	left	-32	-84	-36	6.02	<.001	915
	right	4	-68	-20	6.01	<.001	736
**DEACTIVATIONS**							
Amygdala	right	24	-4	-22	-4.72	<.001	29
Hippocampus	right	30	-16	-22	-6.14	<.001	82
PHG/Amygdala	left	-16	0	-18	-4.37	<.001	28
Prefrontal cortex							
BA 10	right	6	56	-6	-5.03	<.001	138
BA 45	left	-42	22	20	-5.12	<.001	60
BA 6	left	-52	-4	28	-5.85	<.001	74
	right	22	-18	62	-4.44	<.001	31
BA 8	left	-12	44	42	-5.69	<.001	44
BA 9	right	6	48	20	5.36	<.001	55
Cingulate cortex							
BA 25	left	0	4	-26	-4.25	<.001	46
	right	2	28	-18	-6.57	<.001	392
BA 31	right	24	-60	16	-4.43	<.001	32
Parietal cortex							
BA 40	left	-42	-32	46	-6.03	<.001	355
BA 40	right	36	-42	56	-4.28	<.001	60
BA 3	left	-50	-14	54	-4.53	<.001	53
BA 7	left	-28	-52	58	-4.99	<.001	79
	right	16	-54	58	-6.29	<.001	42
Temporal cortex							
BA 20	left	-64	-52	-18	-5.50	<.001	80
	right	44	-18	-30	-5.51	<.001	85
BA 21	left	-60	-48	6	-4.21	<.001	28
	right	66	-42	-16	-5.63	<.001	64
BA 22	left	-58	-2	-4	-6.96	<.001	527
	right	58	-24	2	-5.76	<.001	35
BA 36	left	-30	-32	-22	-5.36	<.001	290
	right	26	-36	-16	-4.34	<.001	47
BA 38	left	-26	12	-42	-6.69	<.001	417
	right	52	8	-24	-5.94	<.001	245
BA 39	right	36	-70	34	5.15	<.001	80
BA 41	left	-56	-24	8	-4.95	<.001	35
Visual cortex							
BA 17	left	-24	-98	-14	-7.91	<.001	180
BA 18	left	-14	-82	22	-5.35	<.001	94
	right	6	-76	2	-6.15	<.001	175
BA 19	left	-44	-74	-12	-7.88	<.001	515
	right	48	-76	-2	-6.53	<.001	418

Abbreviations: BA, Brodmann area; PHG, parahippocampal gyrus.

### Structural equation modeling (SEM)

A corticolimbic network previously shown to be engaged in the response to aversive visceral stimulation and potentially altered by 5-HTTLPR polymorphism was tested using SEM. As described in the methods, nodes of the network were chosen from the task PLS analysis. The coordinates of the chosen seeds are given in Table 2. Chi-squared tests of group differences were performed for baseline and for inflation. The path coefficients and chi-square statistics are shown in Tables 3 and 4. Differences in the magnitude of the path coefficient reflect increased or decreased coupling between the regions while a difference in sign reflects a reversal or qualitative change in regional interactions.

**Table 2:** Location of seed voxels for SEM.

Region	X	Y	Z
Amygdala	24	-4	-22
Hippocampus	30	-16	-22
sgACC	2	28	-18
pACC	2	32	20
mPFC	16	54	6
vlPFC	30	38	-4
dlPFC	48	46	18

Abbreviations: sgACC, subgenual cingulate cortex; pACC, pregenual anterior cingulate cortex; mPFC, medial prefrontal cortex; vlPFC, ventro-lateral prefrontal cortex; dlPFC, dorsolateral prefrontal cortex

**Table 3:** SEM path coefficients and chi-square statistics during baseline.

Baseline			*s/s*			*l*-carrier			λ^2^_diff_
			Path coeff	S.E.	P	Path coeff	S.E.	P	
mPFC	<—	vlPFC	-0.325	0.233	0.163	-0.036	0.284	0.898	0.6
pACC	<—	sgACC	0.154	0.302	0.61	0.399	0.280	0.154	1
**amyg**	**<—**	**pACC**	-0.398	0.212	0.061	0.032	0.188	0.865	**3.9**
sgACC	<—	amyg	-0.475	0.395	0.229	0.46	0.455	0.312	3.3
dlPFC	<—	hippo	0.076	0.145	0.601	0.074	0.495	0.881	0
amyg	<—	hippo	0.033	0.156	0.833	-0.272	0.193	0.16	2.8
hippo	<—	dlPFC	1.192	0.746	0.11	0.287	1.751	0.87	1.2
**mPFC**	**<—**	**dlPFC**	-0.464	0.37	0.21	1.224	0.718	0.088	**9.9**
pACC	<—	mPFC	0.438	0.256	0.087	-0.02	0.277	0.943	2.7
amyg	<—	mPFC	0.186	0.212	0.379	0.333	0.206	0.107	0.6
hippo	<—	amyg	-0.679	0.706	0.337	-0.668	0.583	0.252	0

mPFC, medial prefrontal cortex; vlPFC, ventro-lateral prefrontal cortex; pACC, pregenual anterior cingulate cortex; sgACC, subgenual cingulate cortex; amyg, amygdala; hippo, hippocampus; dlPFC, dorsolateral prefrontal cortex

λ^2^_diff_ > 3.84 is considered significant and is bolded in the table.

**Table 4:** SEM path coefficients and chi-square statistics during inflation.

Inflation			*s/s*			*l*-carrier			λ^2^_diff_
			Path coeff	S.E.	P	Path coeff	S.E.	P	
mPFC	<—	vlPFC	-0.011	0.139	0.936	-0.135	0.143	0.347	0.3
pACC	<—	sgACC	0.279	0.231	0.227	0.25	0.26	0.335	0
amyg	<—	pACC	0.075	0.291	0.797	0.706	0.418	0.091	3.3
sgACC	<—	amyg	0.432	0.379	0.254	-0.159	0.347	0.647	3.4
dlPFC	<—	hippo	-0.151	0.231	0.512	-0.114	0.449	0.8	0
**amyg**	**<—**	**hippo**	0.412	0.315	0.19	-0.458	0.364	0.208	**6.1**
hippo	<—	dlPFC	-0.867	0.533	0.104	-0.384	0.654	0.558	0.7
mPFC	<—	dlPFC	0.412	0.257	0.109	0.066	0.257	0.799	2.2
pACC	<—	mPFC	-0.318	0.376	0.397	-0.611	0.45	0.175	0.8
**amyg**	**<—**	**mPFC**	-0.222	0.405	0.584	1.854	0.665	0.005	**18.9**
hippo	<—	amyg	-0.47	0.387	0.224	0.494	0.304	0.104	3.7

mPFC, medial prefrontal cortex; vlPFC, ventro-lateral prefrontal cortex; pACC, pregenual anterior cingulate cortex; sgACC, subgenual cingulate cortex; amyg, amygdala; hippo, hippocampus; dlPFC, dorsolateral prefrontal cortex

λ^2^_diff_>3.84 is considered significant and is bolded in the table.

During baseline, male subjects with *s/s* genotype demonstrated a significantly increased negative influence of pregenual ACC (pACC) on amygdala activity compared to *l*-carriers. In addition, male subjects with *s/s* genotype demonstrated a weak negative influence of dorsolateral prefrontal cortex (dlPFC) on medial PFC (mPFC) activity while *l*-carriers demonstrated a strong positive influence of dlPFC on mPFC activity.

**Fig 1:**
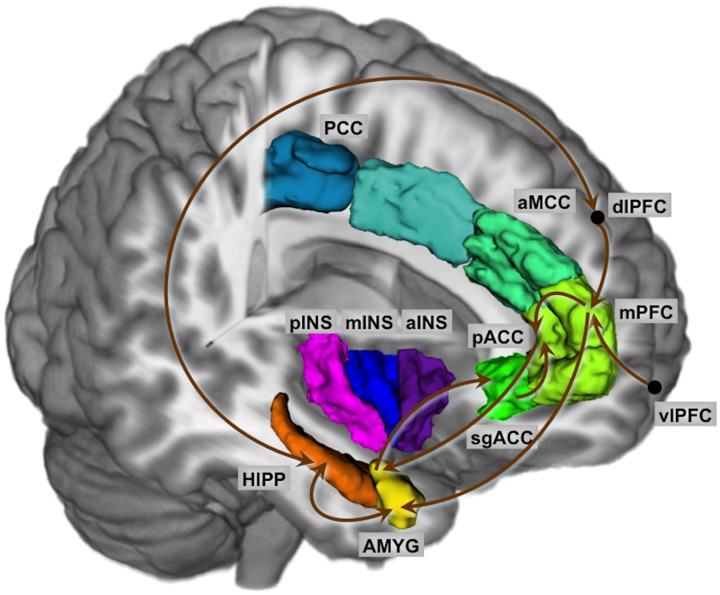
Connectivity Model for Baseline and Colorectal Distention. Nodes of the network were chosen from the task PLS analysis. Regions: AMYG, amygdala; HIPP, hippocampus, sgACC, subgenual cingulate cortex; pACC, pregenual anterior cingulate cortex; mPFC, medial prefrontal cortex; dlPFC, dorsolateral prefrontal cortex; vlPFC, ventro-lateral prefrontal cortex; aMCC, anterior mid cingulate cortex; PCC, posterior cingulate cortex; aINS, anterior insula; mINS, mid insula; pINS, posterior insula.

**Fig 2:**
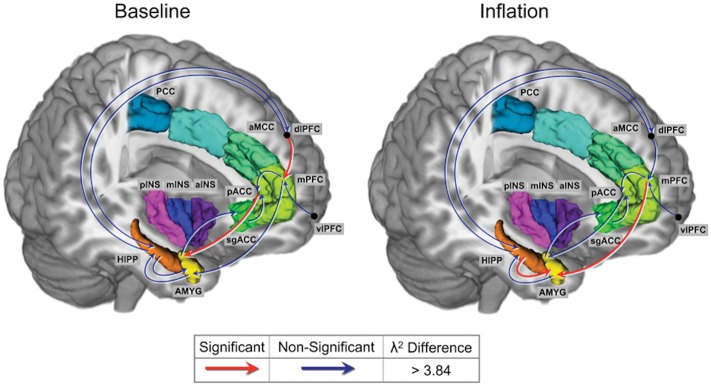
Connectivity Analysis for Baseline and Colorectal Distention. Chi square difference statistics during baseline and colorectal distention. λ^2^ difference > 3.84 is considered significant and is denoted in red. λ^2^ difference < 3.84 is considered non-significant and is denoted in blue. Regions: AMYG, amygdala; HIPP, hippocampus, sgACC, subgenual cingulate cortex; pACC, pregenual anterior cingulate cortex; mPFC, medial prefrontal cortex; dlPFC, dorsolateral prefrontal cortex; vlPFC, ventro-lateral prefrontal cortex; aMCC, anterior mid cingulate cortex; PCC, posterior cingulate cortex; aINS, anterior insula; mINS, mid insula; pINS, posterior insula.

During inflation, male subjects with *s/s* genotype demonstrated a significantly greater positive influence of hippocampus on amygdala activity compared to *l*-carriers. In addition, subjects with *s/s* genotype demonstrated a weak negative influence of mPFC on amygdala activity while *l*-carriers demonstrated a strong positive influence of mPFC on amygdala activity. Fig 1 and Fig 2 summarize the results from the connectivity analysis.

## Discussion

In this sample of healthy male Japanese subjects, individuals carrying the *s/s* genotype demonstrated greater engagement of a network containing pain and emotion-related regions during aversive rectal inflation compared to *l*-carriers. Specifically, *s/s* genotype showed greater activation of sensory, sensory integration and lateral prefrontal regions within this network, and greater deactivation of affect related regions. Furthermore, *s/s* genotype subjects demonstrated altered connectivity, both at baseline and during inflation, in a corticolimbic network engaged in response to aversive visceral stimulation. During baseline, *s/s* subjects demonstrated more negative influence of pACC on amygdala activity, and more negative influence of dlPFC on mPFC activity, expected to result in greater corticolimbic inhibition. During inflation, *s/s* individuals displayed a more “negative” influence of mPFC activity on amygdala activity, and a more positive influence of hippocampal activity on amygdala activity compared with male *l* carriers. In the following we will discuss these findings in the context of the literature and possible clinical implications.

### Greater responsiveness of distension related network in *s/s* individuals

Men with *s/s* genotype showed greater engagement of visceral afferent processing network, which included increased activation of regions of a sensory network (including bilateral thalamus, right anterior/mid insula, basal ganglia) and increased activation of prefrontal and parietal cortical regions. The involved brain regions are consistent with a quantitative meta-analysis of brain regions activated during rectal distension [33]. However, the results highlight different aspects of 5-HTTLPR genotype influence on the brain response to aversive visceral stimulation compared to a previous report [16]. In the previous report, subjects (a heterogeneous sample, of predominantly healthy male subjects) with *s/s* genotype displayed greater activation of the right hippocampus and left ACC during 40 mmHg inflation relative to baseline (0 mmHg) in a standard univariate ANOVA analysis. The current results from the PLS analysis (a more data-driven method) in a homogeneous sample of healthy male subjects, demonstrates that a greater proportion of the variance in the data is accounted for by a pattern of greater activation in sensory and cognitive regions [24] and greater deactivation in affective regions in males with *s/s* genotype. In comparison to the previous report, a more ventral portion of the right hippocampus is reported as a reliable member of a network deactivated to a greater extent in males with *s/s* genotype. Thus, while a more regional approach previously highlighted greater pain response in emotion-related regions in *s/s* genotype subjects relative to *l*-carrier individuals, the current analysis suggests that males with *s/s* genotype have a widespread emotion-related network deactivation to a greater extent during a visceral nociceptive stimulus.

Our present data of more deactivation of emotion-related network in Japanese males are at least in part contradictory to the earlier findings from European descent samples [10, 11]. Hariri et al. [10] reported that female dominant individuals with the *s* allele of 5-HTTLPR exhibit greater amygdala neuronal activity in response to (visual) fearful stimuli than individuals homozygous for the *l* allele. Pezawas et al. [11] also demonstrated tight coupling as a feedback circuit implicated in the extinction of negative affect by functional analysis of the ACC and amygdala during perceptual processing of fearful visual stimuli and relative uncoupling of this circuit in *s* allele carriers. Precise reasons for the discrepancy between our findings and the prior findings is unknown, but may be attributable to different tasks (aversive visceral stimulus vs. emotional recognition task) and/or different genetic backgrounds of the study subjects (Japanese vs European descent). Actually there is a report of more deactivation of pACC in response to esophageal distention in European descent individuals with *s/s* genotype than those with *l* allele [34], which is in the same direction of 5-HTTLPR influence on brain response to visceral stimulation. It is of interest to consider culture-gene coevolution of individualism-collectivism and 5-HTTLPR. Chiao and Blizinsky [35] found evidence that collectivistic cultures were significantly more likely to comprise individuals carrying the *s* allele of the 5-HTTLPR across 29 nations. Not only 5-HTTLPR but also other neurotransmitter genes interact with ecological and social factors to influence psychocultural differences [36]. The functional utility of endophenotypes associated with the 5-HTTLPR may systematically vary as a function of environmental and cultural context. More deactivation of emotion-related network in Japanese males with *s/s* genotype may be driving the lower prevalence rates of anxiety disorders and depressive disorders in Japan, where the genetic pool of *s* allele is greater.

### Greater engagement of corticolimbic circuits in *s/s* individuals

During baseline, *s/s* subjects demonstrated more negative influence of pACC on amygdala activity. This may be partially due to increased resting-state rCBF in amygdala in *s/s* males [37] and the inhibitory effect of pACC on the amygdala [11]. During baseline, *l* carriers had more positive influence of dlPFC on mPFC activity than *s/s* subjects. During rectal distention, *l* carriers had more positive influence of mPFC on amygdala activity than *s/s* subjects. This is consistent with the findings in the earlier report [11]. Thus, *l* carriers can more effectively regulate amygdala activity via the dlPFC-mPFC interactions than *s/s* subjects. Subjects with *s/s* genotype demonstrated a greater positive influence of hippocampus on amygdala activity during inflation while *l* carriers had a negative influence of hippocampus on amygdala activity. The pattern of functional connectivity between the hippocampus, mPFC and amygdala in this study is consistent with data that highlights the role that the hippocampus plays in encoding contextual memories, and suggests that hippocampal-mPFC-amygdala circuits mediate contextual retrieval of fear memories after extinction in rats and humans [38]. The hippocampus projects directly to the basolateral nucleus of amygdala (BLA), and this projection may be crucial for the renewal of fear expression in response to an extinguished conditional stimulus. Indirect projections between the hippocampus and amygdala via the mPFC might also mediate the context-dependent expression of fear in response to an extinguished conditional stimulus. In particular, prelimbic mPFC projections in rodents to the BLA are involved in fear renewal, whereas infralimbic mPFC projections to intercaleted cells, which in turn inhibit central nucleus of the amygdala (CeA) output, are involved in suppressing the expression of fear in response to an extinguished conditional stimulus [39]. Moreover, more “positive” amygdala-prefrontal connectivity has been previously associated with better emotion regulation performance [40]. Our results and reported data suggest that the essential role of 5-HTTLPR in serotonergic neurotransmission and direction of connectivity among the brain regions are common regardless of culture and ethnicity and that 5-HTTLPR may affect positive or negative influence on amygdala activity as well as aversive emotional memory depending on the “environmental context” including culture.

The serotonin system within the amygdala and mPFC play an important role in regulating stress coping behavior [41]. Serotonin activation of 5-HT_3_ receptors on γ-aminobutyric acid (GABA)ergic interneurons innervating the amygdala exerts an inhibitory influence on amygdala activity [41]. The role of the amygdala in the regulation of pain is dual, varying from pronociception to antinociception [42]. For example, it has been shown that serotonin mediated activation of metabotropic glutamate receptors 8 can inhibit nociceptive behavior with an increase in 5-HT and glutamate release, a decrease in GABA, and the inhibition of pronociceptice ON-cell and the stimulation of antinociceptive OFF-cell activities [42]. Therefore, differences in the connectivity of the corticolimbic circuit between *s/s* individuals and *l* carriers may be due in part to sustained neurotransmission of 5-HT and the other (e.g. glutamate, GABA, etc.) neurotransmitters.

### Limitations

There are several limitations to this study, including small sample size and the failure to take into account epigenetic influences and epistatic effects of other genes. In addition, there is a difference in treating heterozygosity of 5-HTTLPR between our study and earlier well-known studies. Earlier studies compared the *l/l* genotype versus the *s/l* and *s/s* genotypes based on the hypothesis that the *s* allele is dominant [10, 11]. We compared the *s/s* genotype versus the *l* allele because the *s* allele is more frequent in the Asian population [12, 15]. Moreover, even in European descent studies, others reported the *l* allele to be dominant [3, 43, 44]. Fourth, molecular mechanism of difference in connectivity between *s/s* genotype subjects and *l*-carrier is still unknown. However, lower 5-HT1A binding was found in *s*-carrier primates throughout both cortical brain regions and the raphe nuclei [45]. The underlying serotonin neurochemical system may be influenced by this functional polymorphism and illustrate the strong potential for extending the nonhuman primate model into investigating the role of this genetic variant on behavior and gene-environment interactions. Despite these limitations, the present study clearly demonstrated the effect of 5-HTTLPR on interoceptive emotional processing.

### Possible clinical implications

IBS is a common, stress sensitive disorder presenting with chronically recurring abdominal pain associated with altered bowel movements and increased anxiety [46]. In healthy European descent females, acute tryptophan (precursor of 5-HT) depletion, presumably resulting in acute reduction in synaptic 5-HT concentrations, was found to be associated with increased connectivity between amygdala and other nodes of emotional arousal and homeostatic afferent networks, and loss of negative feedback inhibition of the amygdala by the ACC during colorectal distention [2], a pattern similar to that observed in female European descent IBS patients [27], and consistent with increased emotional arousal. Although a meta-analysis failed to identify significant associations between 5-HTTLPR and IBS in either European descents or Asians [47], 5-HTTLPR may influence on IBS related endophenotypes [48]. Some reports suggest that the *s* allele of 5-HTTLPR is associated with pain sensitivity [49] and brain response to colorectal distention [16] in mixed sample of IBS patients and controls and pain severity in IBS patients [49]. In support of the hippocampal-amygdala connectivity and pain perception described above, reduced hippocampal glutamate levels measured with magnetic resonance spectroscopy in IBS patients have been reported [50]. Furthermore, the findings in this study would be greatly enhanced if more genetic variations associated with different responses upon visceral stimuli are found with replication of 5-HTTLPR.

In conclusion, the present data suggest that individuals with a weak function of serotonin transporter respond to gut signals differently in the brain regions from those with a strong function of serotonin transporter. Further studies on influence of functional gene polymorphism on long-lasting neural processing whose signal is originated from the gastrointestinal tract are warranted.
